# Mannose-binding lectin 2 (*Mbl2*) gene polymorphisms are related to protein plasma levels, but not to heart disease and infection by *Chlamydia*

**DOI:** 10.1590/1414-431X20165519

**Published:** 2016-12-12

**Authors:** M.A.F. Queiroz, S.T.M. Gomes, N.C.C. Almeida, M.I.M. Souza, S.R.C.F. Costa, R.B. Hermes, S.S. Lima, M.M. Zaninotto, M.A.A. Fossa, M.A. Maneschy, R.N. Martins-Feitosa, V.N. Azevedo, L.F.A. Machado, M.O.G. Ishak, R. Ishak, A.C.R. Vallinoto

**Affiliations:** 1 Laboratório de Virologia, Instituto de Ciências Biológicas, Universidade Federal do Pará, Belém, PA, Brasil; 2 Fundação de Hematologia e Hemoterapia do Pará, Belém, PA, Brasil; 3 Hospital de Clínicas Gaspar Vianna, Belém, PA, Brasil; 4 Hospital Beneficência Portuguesa, Belém, PA, Brasil

**Keywords:** Mannose-binding lectin, Polymorphisms, Heart disease, Chlamydia

## Abstract

The presence of the single nucleotide polymorphisms in exon 1 of the *mannose-binding lectin 2* (*MBL2*) gene was evaluated in a sample of 159 patients undergoing coronary artery bypass surgery (71 patients undergoing valve replacement surgery and 300 control subjects) to investigate a possible association between polymorphisms and heart disease with *Chlamydia* infection. The identification of the alleles *B* and *D* was performed using real time polymerase chain reaction (PCR) and of the allele *C* was accomplished through PCR assays followed by digestion with the restriction enzyme. The comparative analysis of allelic and genotypic frequencies between the three groups did not reveal any significant difference, even when related to previous *Chlamydia* infection. Variations in the MBL plasma levels were influenced by the presence of polymorphisms, being significantly higher in the group of cardiac patients, but without representing a risk for the disease. The results showed that despite *MBL2* gene polymorphisms being associated with the protein plasma levels, the polymorphisms were not enough to predict the development of heart disease, regardless of infection with both species of *Chlamydia*.

## Introduction

Several infectious agents represent important risk factors in the development of atherosclerosis (1,2); among them, *Chlamydia pneumoniae* in endothelial tissue has been strongly associated with coronary artery disease (CAD) (3–6). Persistent *C. pneumoniae* infection may contribute to the development of atherosclerosis by stimulating the local immune response, possibly by triggering the chronic activation of inflammatory pathway components (7–9).

Mannose-binding lectin (MBL) is an important serum protein related to the innate immunity. It binds to mannose carbohydrates and N-acetyl glucosamine and is expressed by a wide variety of microorganisms, promoting opsonization, phagocytosis and activation of the complement system (9,10). In the *MBL2* gene exon 1, three non-synonymous single nucleotide polymorphisms (SNPs) were identified. The wild type allele is referred as *A, and the variants are called *B, *C and *D or, collectively, *O (11,12). The MBL*B, MBL*C and MBL*D alleles represent changes in codons 54 (Gli54Asp; SNP ID rs1800450), 57 (Gli57Glu; SNP ID rs1800451) and 52 (Arg52Cis; SNP ID rs5030737), respectively (13). These mutations lead to structural changes in the protein, causing a functional deficiency and a significant reduction in the circulating MBL (14–16). Variations in the *MBL2* gene are responsible for poor opsonization and are associated with increased susceptibility to respiratory infections, including *C. pneumoniae* (17–19).

MBL protein is associated with the prevention of *Chlamydia* infection. The 40 kDa glycoprotein carbohydrate of *C. trachomatis* and *C. pneumoniae*, known to mediate bacteria attack and infectivity in the host cell membrane, seems to play the role of a ligand for MBL (20). According to Swanson et al. (21), MBL has the potential to inhibit infection of certain cell types by different *Chlamydia* species, suggesting a protective role against these bacteria. However, certain polymorphisms in the *MBL2* gene have been associated with increased risk of *Chlamydia* infection (22).

*C. pneumoniae* infection appears to promote the development and progression of serious CAD (4), especially in patients with *MBL2* gene mutations (7). MBL-deficient patients may present an earlier onset of atherosclerosis or a more rapid disease progression than patients without deficiency in the protein (23). It has also been observed that individuals with at least one mutant allele (MBL2*O) have a carotid plaque area (CPA) – an intermediate atherosclerosis phenotype – significantly larger and more dispersed than that of homozygous individuals for MBL*A allele (24).

Considering the functional role of MBL in the immune response of the human host, the present study aimed to compare the frequency of allelic variants in *MBL2* gene exon 1 between groups of patients with different heart diseases and healthy control subjects, investigating the possible association of SNPs in the *MBL2* gene and changes in MBL plasma levels, in association with *Chlamydia* infection.

## Patients and Methods

### Subjects

This was a cross-sectional case-control study. The population included 159 patients with CAD with the indication for coronary artery bypass graft (CABG) surgery and another group of 71 patients with heart valve disease (HVD) who presented surgical indications for prosthetic valve implant (mitral or aortic valve replacement). Patient inclusion criteria were individuals of both sexes, aged over 18 years, admitted with indications for a surgical procedure for the first time and who were not taking antibiotics.

Samples were collected between November 2010 and July 2012 in the Hospital Beneficência Portuguesa, the Hospital da Ordem Terceira, and the Fundação Hospital das Clínicas Gaspar Viana, all located in the city of Belém, PA, Brazil. A healthy control group (CG) of 300 blood donors from the Fundação Centro de Hemoterapia e Hematologia do Pará (HEMOPA) without diagnosis of heart disease had demographic information and serum samples collected to compare the frequency of polymorphisms, plasma levels and cytokine gene expression. The control group was matched by sex and age with the group of cardiac patients.

### Specimen collection

A blood sample (10 mL) was collected from patients and controls by intravenous puncture using a vacuum collection system containing EDTA as an anticoagulant. The samples were separated into plasma and leukocytes. Plasma was used for the detection of antibodies to *C. trachomatis* and *C. pneumoniae*, and MBL plasma levels. Leukocyte samples were used for genomic DNA extraction and for the analysis of genetic polymorphisms of *MBL2* exon 1. Plasma and leukocyte samples were stored at -20°C until the time of use. The project was submitted to and approved by the HEMOPA Research Ethics Committee (Case #0011.0.324.000-09). All participants were properly informed about the research objectives, and those who accepted to take part, signed an informed consent form.

### Detection of *Chlamydia* antibodies

Antibodies were detected using an enzyme immune assay (ELISA) for the detection of anti-*C. trachomatis* (NovaLisa TM *Chlamydia trachomatis* IgM and IgG, Germany) and anti-*C. pneumoniae* (NovaLisa TM *Chlamydia pneumoniae* IgM and IgG), as established by the manufacturer.

### DNA extraction

DNA extraction from peripheral blood leukocytes used phenol-chloroform (25), and the procedure followed cell lysis, protein and DNA precipitation and hydration.

After extraction, the DNA obtained was quantified using a Qubit^®^2.0 fluorometer (Life Technologies, USA) and Qubit^™^ DNA assay kit (Life Technologies) solutions, following the manufacturer's recommended protocol.

### Genotyping MBL2 rs1800450 (MBL*B) and rs5030737 (MBL*D)

The analysis of polymorphic alleles MBL*B and MBL*D, used a real-time PCR performed on the StepOnePLUS^™^ real-time PCR system. Two commercial TaqMan^®^ SNP Genotyping Assays (Life Technologies) were used: PartNumber C_2336609_20 for identification of the allele MBL*B (rs1800450) and C_2336610_10 for identification of the allele MBL*D (rs5030737). The cycling conditions were as follows: 40 cycles of 10 min at 95°C, 15 s at 95°C, and 1 min at 60°C.

### Genotyping MBL2 rs1800451 (MBL*C)

MBL*C polymorphism analysis was performed by PCR to amplify a 120-bp segment of the *MBL2* gene exon 1 (26). Amplifications were performed on a Peltier Thermal Cycler equipment (Biocycler, USA) in a final volume of 50 µL, containing 500 ng of total extracted DNA, 225 µM of each dNTP, 5 µM of each primer, 1.1 mM MgCl_2_, 50 mM KCl, 10 mM Tris-HCl, pH 8.3, and 0.5 U of Taq DNA polymerase (Invitrogen, USA) and the following primer pair: (mblE01) 5′-AGTCGACCCAGATTGTAGGACAGAG-3′ and (mblE02) 5′-AGGATCCAGGCAGTTTCCTCTGGAAGG-3′. In each amplification reaction after an initial denaturation at 94°C for 5 min, 35 cycles were performed of 30 s at 94°C (denaturation), 1 min at 58°C (annealing), 2 min at 72°C (extension), and a final extension of 10 min at 72°C. The identification of the MBL*C allele was performed by polymorphism analysis with restriction enzymes (RFLP) using the Mbo II enzyme. The PCR-RFLP reaction products were visualized using 4% agarose gel electrophoresis (100 V/45 min) in 1x TAE buffer (TAE 40 x stock, 1.6 M TrisBase, 0.8 M sodium acetate, and 40 mM Na_2_-EDTA/1000 mL of deionized water) containing 6 µL of SYBR^®^ Safe DNA gel staining (10 mg/mL; Invitrogen), on a transilluminator with an ultra-violet light source.

### MBL plasma level dosage

MBL plasma concentration was evaluated in 230 patients (159 with CAD and 71 with HVD) and 250 control subjects using an ELISA test following manufacturer's recommended technical procedures (Human MBL Quantikine ELISA Kit, R & D system, USA).

### Statistical analysis

The genotypic and allelic frequencies were estimated using direct counting, and differences between the groups were compared using the chi-square and G tests. A Hardy-Weinberg equilibrium calculation was performed to assess the distributions of observed genotype frequencies. Analysis of serum MBL level was performed using the Kruskal-Wallis and Mann-Whitney nonparametric tests; the genotypes carrying mutations *B, *C, and *D were grouped as AO or OO to minimize deviations resulting from the small sample sizes. All tests were performed using the BioEstat 5.3 program (Brazil) (27), and associations with a value of P<0.05 were considered to be significant.

## Results

Genotype AA of the *MBL2* gene was the most common, but no significant differences were observed in the genotypic and allelic frequencies between the three groups investigated (Table 1), even when comparing the presence of markers of past infections by *C. trachomatis* and *C. pneumoniae* (Table 2).

**Table:**
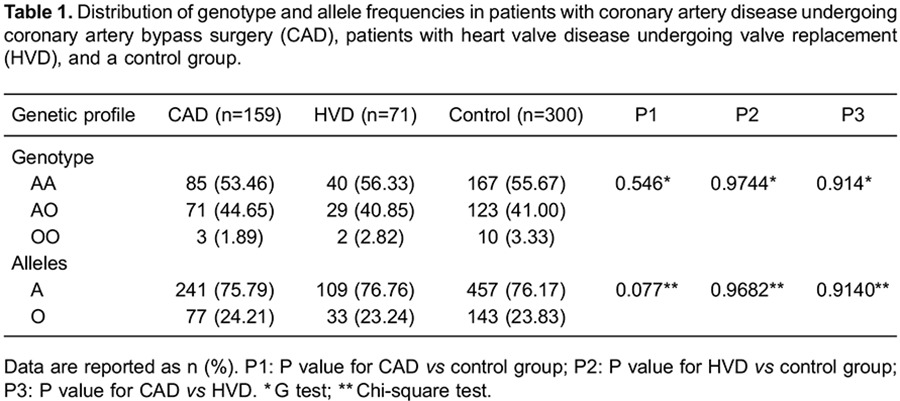

**Table:**
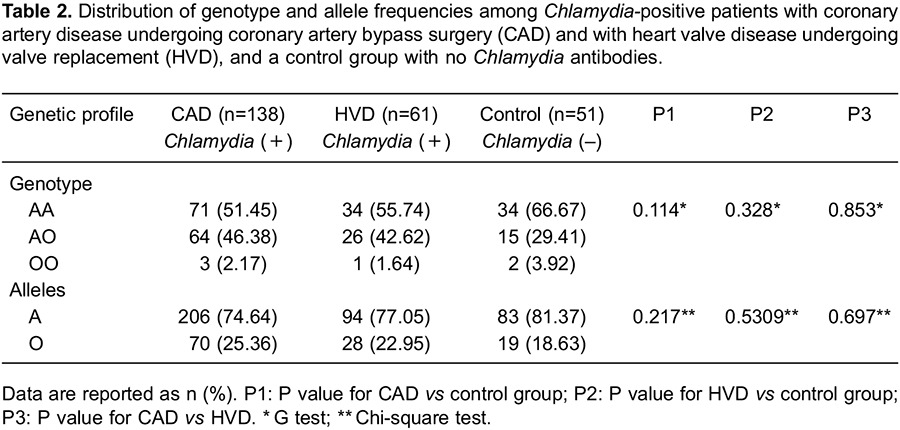

**Figure 1:**
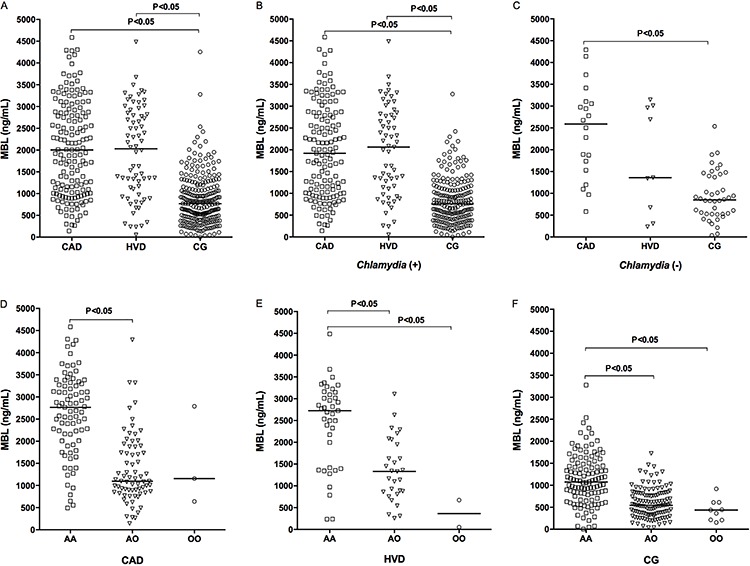
Distribution of mannose-binding lectin (MBL) plasma levels in patients with coronary artery disease undergoing coronary artery bypass surgery (CAD), patients with heart valve disease undergoing valve replacement (HVD), and the control group (CG) (*A*); among the three groups with positive (*B*) and negative serology (*C*) for *Chlamydia,* and in relation to the different genotypes of each group (*D*, *E*, and *F*). Data are reported as medians (Kruskal-Wallis and Mann-Whitney tests).

The median values of MBL plasma level were compared, showing that in the patient groups (CAD and HVD) there were significantly higher protein concentrations than in the control subjects (Figure 1A). A similar pattern in the distribution of MBL levels was observed in the three groups seropositive for the genus *Chlamydia* (Figure 1B).

Comparison of the groups without evidence of prior *Chlamydia* infection revealed significant differences in the plasma levels between the CAD and control groups (Figure 1C). According to the different genotypes in the *MBL2* gene exon 1, individuals with the AA homozygous wild type genotype had significantly higher plasma MBL levels than those with AO and OO genotypes, among the CAD, HVD and CG groups (Figure 1D, E, and F).

**Figure 2:**
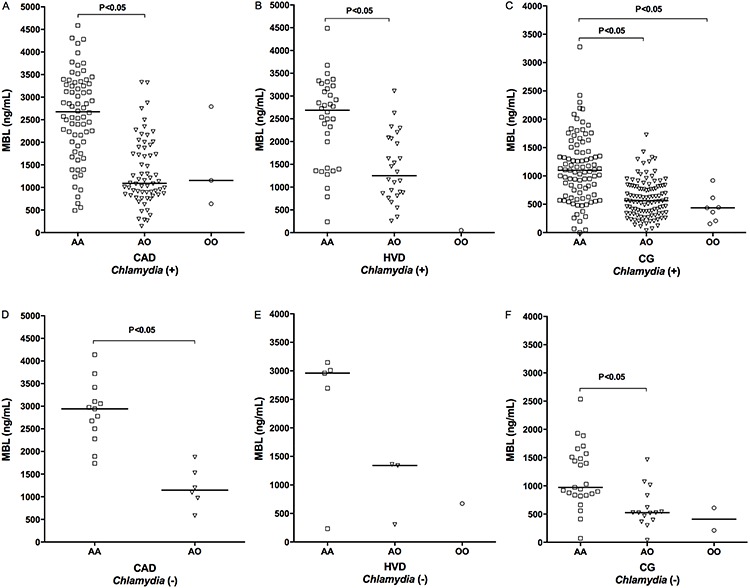
Distribution of mannose-binding lectin (MBL) plasma levels in relation to the different genotypes of (*A*) patients with coronary artery disease undergoing coronary artery bypass surgery (CAD), (*B*) patients with heart valve disease undergoing valve replacement (HVD), and (*C*) control subjects (CG) seropositive for *Chlamydia,* and seronegative for *Chlamydia* (*D*, *E* and *F*). Data are reported as medians (Mann-Whitney test).

Comparative analysis of the MBL plasma levels between genotypes for the variations located on the *MBL2* gene intron 1, according to previous seropositivity in the three groups, demonstrated that individuals with an AA genotype had significantly higher MBL levels than individuals with at least one allele variant (Figure 2A, B, and C). However, individuals without evidence of prior *Chlamydia* infection in the CAD and CG groups carriers of the AA genotype had significantly higher MBL plasma levels than those who had a heterozygous genotype for the investigated variations (Figure 2D and F). The same analysis in patients of the HVD group revealed no significant differences (Figure 2E).

**Figure 3:**
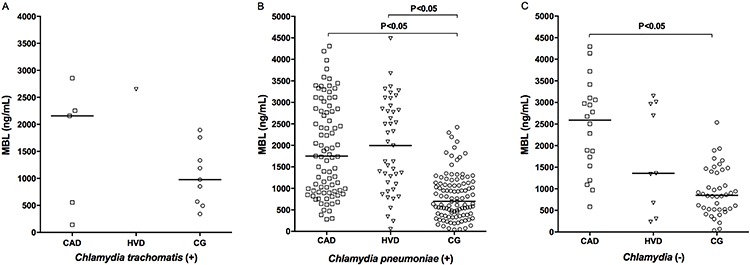
Distribution of mannose-binding lectin (MBL) plasma levels in patients with coronary artery disease undergoing coronary artery bypass surgery (CAD), patients with heart valve disease undergoing valve replacement (HVD) and a control group (CG) seropositive only for *C. trachomatis* (*A*), seropositive only for *C. pneumoniae* (*B*), and seronegative for *Chlamydia* (*C*). Data are reported as medians (Mann-Whitney test).

MBL plasma levels between groups (CAD, HVD, and CG) were evaluated according to the presence of serological markers for the *Chlamydia* species and were compared with the groups that did not show evidence of prior infection to either species. Participants who were seropositive only for *C. trachomatis* showed no significant difference in the MBL plasma levels among groups (Figure 3A). A significantly higher MBL levels was observed among CAD and HVD patients seropositive for *C. pneumoniae* compared to the control group (Figure 3B). Analysis of participants without evidence of prior *Chlamydia* infection showed significantly higher MBL plasma levels in CAD patients compared with those of the CG (Figure 3C).

**Figure 4:**
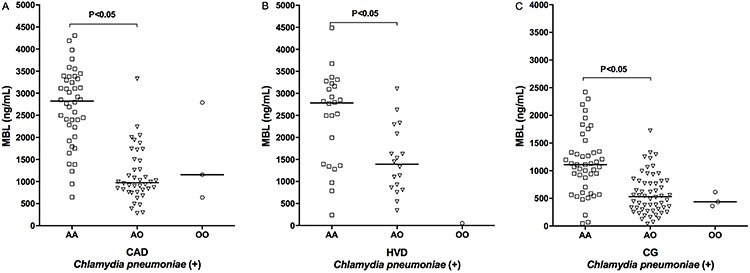
Distribution of mannose-binding lectin (MBL) plasma levels in relation to the genotypes of (*A*) patients with coronary artery disease undergoing coronary artery bypass surgery (CAD), (*B*) patients with heart valve disease undergoing valve replacement (HVD), and (*C*) control group individuals (CG) seropositive only for *C. pneumoniae*. Data are reported as medians (Mann-Whitney test).

As a consequence of the small number of individuals in the CAD, HVD and CG groups carrying *MBL2* gene mutations and serological evidence of past infection with *C. trachomatis*, it was not possible to perform a comparative analysis, but individuals carrying *MBL2* gene mutations who were previously infected with *C. pneumoniae* revealed significant differences in the MBL plasma levels among the *AA* and *AO* genotype carriers for all three investigated groups (Figure 4A, B, and C).

## Discussion

In the present study, we investigated the possible association between the presence of *MBL2* gene exon 1 mutations and previous infections with two species of *Chlamydia* in the predisposition for the development of cardiovascular disease. Variations in the *MBL2* gene, which induce low serum protein levels, are associated with the susceptibility to infection and appear to influence the development of CAD (7,18,24).

Madsen et al. (23) observed that the homozygous genotype encoding *MBL2* deficiency could be considered a risk factor for early onset of atherosclerosis or, sometimes, a more intense progression of the disease. It was observed that functionally deficient *MBL2* variants were associated with a doubled risk of myocardial infarction and an increase in atherosclerosis (28). Variations in the promoter region associated with variations in the *MBL2* gene exon 1 predictive of lower MBL levels were significantly related to CAD, regardless of other risk factors (18). The results of these studies showed that the allele determining high MBL levels in patients with rheumatoid arthritis was considered a risk factor for ischemic heart disease, including myocardial infarction (29). Furthermore, high MBL levels have been associated with an increased risk of developing coronary artery disease in apparently healthy men (30). Elevated MBL levels appear to be related to heart disease, as they allow a greater interaction of the protein with altered endothelial cells, increasing the inflammatory process (31).

The influence of previous *Chlamydia* infection and heart disease, along with the presence of genetic variations in the *MBL2* gene, was initially described by Rugonfalvi-Kiss et al. (7), showing the association of *C. pneumoniae* infection with severe CAD development in patients with allelic variants. In another context, a significant association of a low expression of MBL genotype was further described in cases of damage to the fallopian tube, regardless of the presence of *C. trachomatis* infection (22).

Despite the presence of the AA genotype as the most common genotype in the present study, there was no significant difference in genotype frequencies between the three investigated groups that could imply a relationship of CAD predisposition. Our findings suggest that the presence of genotypes associated to either high or low MBL serum levels were not sufficient to solely act as trustful biomarkers of cardiovascular disease risk in the presence of previous *Chlamydia* infection. However, these results should be further confirmed with a study involving a larger sample size and different population groups, because allele and genotype frequencies are certainly associated with the ethnic profile of the population being studied.

Differences in MBL concentration have been associated with modulation of the immune response to infectious agents and to chronic inflammatory diseases, such as atherosclerosis (8,29,32). In our study, higher MBL plasma levels were observed in patients with cardiovascular disorders (CAD and HVD) than in the CG, in agreement with the evidence showing that high levels of circulating MBL is a risk factor for ischemic diseases, including myocardial infarction (29). The MBL protein recognizes structures exposed in altered endothelial cells, contributing to tissue damage (33,34). In acute myocardial ischemia, MBL inhibition has been shown to reduce the infarction area by promoting a reduction in neutrophil infiltration and pro-inflammatory gene expression (35). Thus, our results suggest that the MBL protein can also be involved in chronic heart disease, similarly to C-reactive protein (CRP), by increasing the inflammatory process, probably via excessive activation of the complement system.

MBL is an important component of the innate immunity, which fights infectious agents and represents the first line of host defense against *Chlamydia* infection (20). Pesonen et al. (8) found a correlation between high *Chlamydia* antibody titers and acute coronary events, which was also associated with high MBL serum levels, demonstrating that despite being related to reduced susceptibility to infection, high MBL levels may also increase the risk of heart disease. However, in our study, previous infections with *C. trachomatis* and *C. pneumoniae* did not affect MBL plasma levels in the 2 groups of patients. This result possibly implies that heart disease, especially atherosclerosis, is multifactorial in its etiology and infection is only one of the components.

MBL levels have been associated with gene variation (29,36,37), and several studies have investigated genotypic variations to predict the levels of this protein and its influence on pathogenic processes (7,22,38). We attempted to investigate both genetic and phenotypic profiles by the identification of polymorphisms in *MBL2* exon 1 and the determination of serum MBL levels. The predictive relationship that high MBL levels are associated with the wild (AA) genotype and low levels are associated with the presence of mutations (AO and OO) was thoroughly confirmed in all the groups. MBL serum levels related to the AA genotype were significantly higher in the cardiac patient groups (CAD and HVD), compared to the controls, suggesting that the presence of wild genotype in patients with inflammatory heart disease induces a sharp increase of the protein synthesis. It should also be noted that all patients, regardless of their genotype, were undergoing surgery; this suggests that alterations in the MBL plasma levels due to the single nucleotide polymorphisms, may not be the sole significant risk factor to be taken into consideration in the evolution of heart disease.

Our results showed that the presence of variants in the exon 1 of the *MBL2* gene are directly associated with MBL plasma levels, but the polymorphisms lack sensitivity to predict the risk for developing heart disease, regardless of a previous infection with *Chlamydia*.
